# On which common ground to build? Transferable knowledge across cases in transdisciplinary sustainability research

**DOI:** 10.1007/s11625-021-01010-0

**Published:** 2021-08-02

**Authors:** Gabriela Wuelser, Carolina Adler, Thomas Breu, Gertrude Hirsch Hadorn, Urs Wiesmann, Christian Pohl

**Affiliations:** 1 USYS TdLab, Institute for Environmental Decisions, ETH Zurich, 8092 Zurich, Switzerland; 2 Mountain Research Initiative, University of Bern, 3012 Bern, Switzerland; 3 Centre for Development and Environment, University of Bern, 3012 Bern, Switzerland; 4 Environmental Philosophy Group, Institute for Environmental Decisions, ETH Zurich, 8092 Zurich, Switzerland

**Keywords:** Knowledge co-production, Transdisciplinarity, Body of knowledge, Transferable knowledge, Sustainability research, Grounded theory, Science studies

## Abstract

To support societal problem solving, transdisciplinary research (TDR) uses knowledge co-production focusing on relevance and validity in a studied case and its particular social–ecological context. In the first instance, the resulting situated knowledge seems to be restricted to these single cases. However, if some of the knowledge generated in TDR could be used in other research projects, this would imply that there is a body of knowledge representing this special type of research. This study used a qualitative approach based on the methodology of grounded theory to empirically examine what knowledge is considered transferable to other cases, if any. 30 leaders of 12 Swiss-based TDR projects in the field of sustainable development were interviewed, representing both academia and practice. The transferable knowledge we found consists of the following: (1) Transdisciplinary principles, (2) transdisciplinary approaches, (3) systematic procedures, (4) product formats, (5) experiential know-how, (6) framings and (7) insights, data and information. The discussion of TDR has predominantly been focusing on transdisciplinary principles and approaches. In order to take knowledge co-production in TDR beyond an unmanageable field of case studies, more efforts in developing and critically discussing transferable knowledge of the other classes are needed, foremost systematic procedures, product formats and framings.

## Introduction

Knowledge co-production is considered a key means to address grand societal challenges (Mauser et al. 2013) and discussed as a way for academia to engage in sustainable development that is worth advancing (Polk 2015). Transdisciplinary research (TDR) is one of the key approaches to knowledge co-production. One of the trendlines of transdisciplinarity prioritizes problem solving for sustainable development (Hadorn et al. 2006). This trendline emerged “in the late 1980s and early 1990s in the Swiss and German context of environmental research” (Klein 2017, 30). Such TDR takes a wicked societal challenge as a point of departure (Rittel and Webber 1973; United Nations 2019). The goal is to provide knowledge–understood in a broad colloquial sense, as the term knowledge co-production implies–that help relevant actors in dealing with that particular societal challenge. In doing so, such TDR aims at grasping the complexity of the issue; taking into account relevant practitioners’ and researchers’ diverse perceptions; linking abstract and case-specific knowledge; and developing descriptive, normative, and transformative knowledge to promote sustainable development (Pohl et al. 2017, 322). To achieve these aims, researchers from different disciplines and actors in other sectors of society jointly go through a TDR process (Jahn et al. 2012; Lang et al. 2012).

To best support societal problem solving, TDR seeks to produce “situated knowledge” (Haraway 1988) that is relevant and valid for a specific case, i.e. a problem situation in its particular social–ecological context. Knowledge produced through TDR, with its focus on relevance and validity for a specific case, may, therefore, not be readily transferable from one project to the next. Nevertheless, transfer of knowledge between cases would be highly relevant for further developing TDR (Nagy et al. 2020). If the generated knowledge is not transferable and every project starts from scratch, TDR will stay a growing but unmanageable field of “jacs” (just another case study). If there is knowledge that can be transferred between cases—implying that there is a body of knowledge in TDR—this would allow for sharing such knowledge among scholars in this field. However, it is not yet clear whether such transferable knowledge exists, and if so, what it consists of.

When studying transferability of knowledge across cases in TDR, one has to account for the fact that co-production of knowledge is conducted by teams of researchers and practitioners, which is different from basic, applied and ideographic research (Adler et al. 2018). Because so many perspectives are involved, TDR processes lead to a bigger variety of outcomes. Literature containing in depth discussions or reports on respective transfers of knowledge used or gained in TDR projects is very rare. Thus, a sufficiently sound basis for investigating this issue is missing. We, therefore, conducted an in-depth qualitative empirical examination to explore issues of knowledge transfer across cases in TDR. For this paper, we focused on the following questions:

1. Via which pathways is knowledge transferred across cases, if at all?
2. What knowledge generated in TDR can be used in other cases?
3. What characterizes transferable knowledge in TDR?

The qualitative research design that we used followed the methodology of grounded theory that dates back to (Glaser and Strauss 1967; Strauss and Corbin 1990). We chose this methodology because in cases where the state of knowledge is insufficient it allows to inductively explore how people perceive and deal with certain issues and potentially find commonalities that may form a basis for a new theory. Thus, we approached the question of transferability of knowledge via experts’ personal and subjective experiences and assessments of such transferability. Our research involved interviews with 30 leaders of 12 Swiss-based TDR projects in the field of sustainable development, representing both science and practice (see Table 1). We chose to interview project leaders because we assumed that they were key persons in defining project design and implementation, and they would potentially decide on knowledge transfer from other cases when starting a new project. We interviewed researchers and practitioners because they collectively develop projects and because we assumed the body of knowledge in TDR to be co-produced by both groups. We studied Swiss-based projects because of the country’s long tradition and experience in TDR for sustainable development (Häberli and Grossenbacher-Mansuy 1998; Klein et al. 2001).

In the following sections, we first present key aspects that underpin a theoretical background for this research. We then describe our research approach and methods more precisely, followed by our findings related to pathways for knowledge transfer, knowledge considered transferable and characteristics of transferable knowledge in TDR. Based on that, we discuss how our findings contribute to understanding and further developing the body of knowledge in TDR, and strengthen co-production of knowledge, more generally. We conclude with pointing out possible limitations of our study.

## Theoretical background

Scientific disciplines use their own, specific definitions of knowledge according to their respective theoretical underpinnings (Becher 1989). The evolving field of TDR does not only span many scientific disciplines, knowledge co-produced in TDR also integrates traditional, indigenous and other forms of non-academic knowledge (Raymond et al. 2010; Tengo et al. 2017). Consequently, knowledge generated in a TDR process encompasses many kinds of sense making, learnings and outcomes (Enengel et al. 2012; Schmidt et al. 2018; Tobias et al. 2019). We thus use the term knowledge in a broad colloquial sense of “understanding of or information about a subject that you get by experience or study, either known by one person or by people generally” (https://dictionary.cambridge.org/de/worterbuch/englisch/knowledge). Since potentially transferable knowledge generated in TDR processes was our object of research, we did not predefine the term knowledge or restrict it to a specific discipline’s understanding. Correspondingly, our starting point was to understand knowledge transferred across cases in TDR as “substantive knowledge derived in one context (case), or methods that have been used to study that case, to another case or type of problem” (Adler et al. 2018, 181). We deliberately excluded knowledge transfer between research and practice (e.g. Roux et al. 2006) from this study. This is because we are interested in knowledge that researchers and practitioners, leading TDR project leaders pass on from one project, or case, to the next. In the terminology of Elkana (1979, 276), such knowledge could constitute the “body of knowledge” of TDR. A body of knowledge encompasses the state of knowledge in a field, with its theories, methods, data, solutions, open questions, conventions and problem framings. A body of knowledge in a given field is what researchers take from one project to the next to develop such knowledge further and it is what experienced scholars impart to newcomers, or, in the longer run, what is published in the academic literature the more a field is established.

Currently, it is not clear to which extent such a body of knowledge in TDR exists and what it entails. In our study, we search for transferable knowledge in TDR by interviewing individuals who have led TDR processes. Some years ago, Gibbons and colleagues suggested such individuals would gather transferable experiences: “Characteristically, Mode 2 research groups are less firmly institutionalised; people come together in temporary work teams and networks, which dissolve when a problem is solved or redefined. […] The experiences gathered in this process create a competence which becomes highly valued and which is transferred to new contexts” (Gibbons et al. 1994, 6). We search for such transferable experiences of individuals, among other things, because we want these experiences, if possible, to become explicit knowledge on how to navigate TDR projects at the level of the wider research community. We assume that the individuals’ transferred knowledge between cases can be a foundation for the body of explicit knowledge and knowing in this group.

## Research design and methods

Considering TDR as an evolving field, and assuming knowledge on transferability as being ‘stored’ in practices, minds and experiences of TDR researchers and practitioners, we conducted a qualitative empirical analysis (Denzin and Lincoln 2005), following the inductive methodology of grounded theory (Corbin and Strauss 2008; Glaser and Strauss 1967). The qualitative approach allowed us to comprehensively take into account the researchers’ and practitioners’ practices, perceptions, experiences and appraisals of knowledge transfer, as well as their underlying reasonings (Denzin and Lincoln 2005). The methodology of grounded theory forced us to stay open for unexpected outcomes and refrain from preconceptions.

### Sample

The sample consisted of 12 recent research projects (Table 1), all of which were at least co-led by academics at Swiss universities. The research work was conducted in various continents, most of it outside Switzerland. When we defined the final sample in 2017, the projects were selected based on whether they had been carried out within the past 5 years. Ongoing projects needed to be at least halfway to completion. All project teams had to work on sustainability problems. Moreover, all projects had to involve both researchers and practitioners.

**Table 1:** Research projects analyzed

Project acronym (number of interviews)	Project goal	Research institution and field	Partner from practice	Country
Glacier hazards GLA (3)	Set up an early warning system for glacier hazards	University of Zurich; glaciology	CARE; Swiss Agency for development and cooperation	Peru
Water governance WGOV (1)	Develop regional water governance options in a mountain area	University of Bern; geography	Various regional municipalities and stakeholders	Switzerland
Climate atlas ATL (3)	Produce a region-specific climate atlas	University of Bern; climatology	University Mayor de San Andres; Bolivian National Service for meteorology and hydrology	Peru, Bolivia
Nuclear waste NUC (3)	Understand why people oppose to nuclear waste repositories	ETH Zurich; environmental sciences	BKW (energy and infrastructure company); National Cooperative for the disposal of radioactive waste	Switzerland
Teenage pregnancy PREG (1)	Understand how girls cope with teenage pregnancy	Swiss TPH; sociology	University of Ghana; Stakeholders from government, funding institutions policymakers, practitioners	Tanzania, Ghana
Low carbon technologies TEC (2)	Identify barriers to integration of low carbon technologies in building design	ETH Zurich; architecture, economics	Tegel Projekt GmbH	Switzerland, Germany
Sanitation design and implementation SAN (2)	Develop a guideline for user driven sanitation design and implementation	Eawag—Swiss Federal Institute of Aquatic Science and Technology; engineering	Asia Development Bank	Thailand, Nepal
Peace building PEACE (2)	Identify potential of private sector engagement to support peace building	Swisspeace (peace research institute); political sciences	Various local private sector companies	Congo
Claims on land LAND (2)	Document multiple claims on land and landscape transformations	University of Bern; geography	Laos Ministry of Agriculture	Laos
Water and land knowledge resources centers WLRC (2)	Establish water and land knowledge resources centers	University of Bern; geography	Ethiopia Water and Land Resource Center	Ethiopia
Water conflicts CON (2)	Develop innovative governance schemes to mitigate water conflicts	University of Bern; geography	Center for Training an Integrated Research in ASAL Development CETRAD	Kenya
Urban planning URB (2)	Develop ‘well-being’ centered urban planning guidelines	University of Geneva; architecture, urban planning	Urban planning consulting firm	Diverse EU countries

Following the principles of qualitative research, we aimed at compiling a heterogeneous sample within the scope of TDR for sustainable development. Such heterogeneity allowed to identify a broad range of knowledge based on a thorough analysis of similarities and differences gained by constant comparison of studied statements (Glaser and Strauss 1967, 110–115). The TDR projects in our sample were selected because they differed with respect to the following: (a) the sustainability problem addressed; (b) geographic or institutional scales (e.g., whether it addressed a local or a national issue); (c) time frames, sizes and number of cases; (d) the conceptualizations of transdisciplinarity, that is, purposes pursued by the project leaders through their model of science-practice collaboration and significance of the research aspect; and (e) the level of TDR expertise of interviewees, ranging from researchers that were rather new in the field of transdisciplinarity to experts with long-standing experience. The latter was important to account for aspects that experienced people may no longer point out.

### Data collection

The main data collection consisted of 25 interviews involving 30 project leaders and co-leaders. Additionally, we consulted written texts of the projects (e.g., reports, web pages) to prepare for and complement the interviews. The interviews were held with project leaders from science and practice. The interviewed practitioners were involved in a project as its co-leading partners, persons with influence in the project, or performing another formative role (beneficiaries of a transdisciplinary project were not interviewed). Academics and practitioners were interviewed separately. The interviews were conducted from 2017 to 2018. All interviews were recorded and transcribed, some transcripts translated into English from Spanish and French, in cases where those interviews were conducted in those languages.

The interviews followed a semi-structured guide. The interviewees were first asked to present a brief project overview and list the main project findings and outcomes. Then they were asked whether anything from what they had listed was or could be used elsewhere. Subsequently, to allow for more in-depth learning, interviewees had to pick one of those (finding, outcome) and were asked about considerations they had made concerning the transferability of their selected item.

### Data analysis

The methodology of grounded theory comprises a series of interconnected procedures for data analysis. In principle, they serve detecting ideas and concepts in the data and checking whether and how they occur across interviews and can be clustered and developed into a set of categories—or eventually even a theory. We limited this study to developing robust classes characterising what the interviewed experts considered transferable in TDR. To analyse the transcribed interview material, all relevant text parts were coded, following the approach of open coding or substantive coding, respectively: “Substantive coding is the process of conceptualizing the empirical substance of the area under study: the data in which the theory is grounded. Incidents are the empirical data (the indicators of a category or concept) from which a grounded theory is generated. The process proceeds from the initial open coding of data to the emergence of a core category, followed by a delimiting of data collection and analysis for selective coding to theoretically saturate the core category and related categories (Holton 2010). Following the principle of constant comparison, we simultaneously conducted text coding, writing memos and paraphrasing summaries, as well as data interpretation in an iterative and recursive process (Corbin and Strauss 2008; Glaser and Strauss 1967). The identified concepts of transferable knowledge were clustered using a categorical mode of thinking approach (Freeman 2017): Repeatedly asking and comparing what the transferable knowledge actually was about led to seven descriptive classes of knowledge. The procedure of constant comparison served controlling for homogeneity of knowledge within classes and selectivity of knowledge between classes. Completeness of classes, however, is typically out of reach for an inductive determination of classes.

In several interviews, we encountered ambiguities of coding, which we dealt with as follows: (a) Some interviewees did not explicitly state what they considered transferable. In some cases, it became clear from the context of the interview transcript text. We only took up information that could be replicated from the original data (i.e., where the contextual information clearly indicated transferability). (b) In some cases, knowledge declared to be transferable represented overall, quite complex approaches or procedures. At the same time, interviewees listed single constituting elements. If the interviewees considered the idea of transferring these single elements valuable, we listed them in the results as well.

## Findings

Our findings are based on 12 thematically different projects. Some of the findings are difficult to understand without linking them to the specific project’s topic. Therefore, for each finding, we indicate the project of origin, using the abbreviations introduced in Table 1. When citing a direct quote, we further differentiate on whether it is a researcher’s (*Res*) or a practitioner’s (*Prac*) statement.

### Pathways of knowledge transfer

**Fig. 1:**
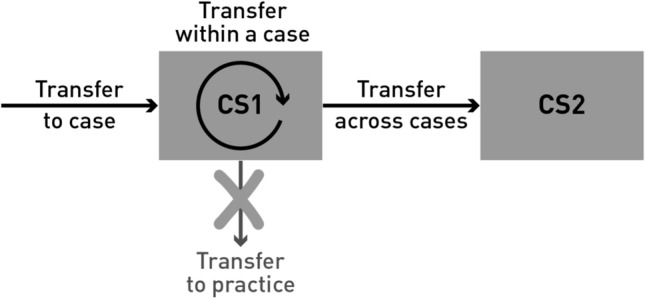
Pathways of knowledge transfer identified and addressed in the study. Knowledge can be transferred across cases: to the studied case, within the studied case or from the studied case to another case. Direct transfer of knowledge to practice was not addressed in this study

The interviewees mentioned several ways for how knowledge was transferred between TDR projects and cases. They can be grouped along three pathways of knowledge transfer (Fig. 1). Independent of the pathway, a transfer was reported to often involve adapting or further developing knowledge derived in an antecedent context for use in a new context.

The first pathway is an input from other, mostly previous research in various fields—not only TDR—to the case of an actual project (*Transfer to case*). In Glacier hazards (GLA), early warning technology was used, which had been previously developed elsewhere (GLA Prac-2 427). The Teenage pregnancy project (PREG) adopted an existing resilience framework to approach teenage pregnancy, not as usually done in the sense of a burden, but from a strength-based perspective. The general idea of developing a climatological atlas used in Climate atlas (ATL) had also existed before, as mentioned by a researcher:

“Well the concept can be used anywhere, and it's also not our invention. Atlases of that sort have begun for other countries. We look at these atlases. We take ideas from these atlases. We are not very close, but we are in discussion with other countries in the region to extend [the] project. We do have an advisory board, and there is, for instance, a German scientist who does similar things in Ecuador.” (ATL Res 40)

An additional example is an approach developed for a transdisciplinary teaching format transferred to research. In this regard, Nuclear waste (NUC) used a specific case study approach to tackling environmental problems. The approach had been developed for a master’s degree course (NUC Res 148).

The second pathway of knowledge transfer functions as a conduit between two projects or project parts operating in parallel on the same case (*Transfer within a case*). Knowledge transfer is thus not restricted to successive projects. For example, in PREG, the research team members conducted the same analysis in two countries in parallel. The ways of dealing with the challenges that emerged during the research were transferred across the different investigated contexts. Thereby, the researchers paid attention to whether the different researchers’ coping strategies could be applied in the other contexts.

The third pathway spans different cases (*Transfer across cases*). Most of the interviewees’ statements on transferable knowledge referred to applying the knowledge developed in the current case to hypothetical future TDR projects that could be comparable to the current case in various ways. A special type of transfer across cases pertains to geographical or temporal extensions, expansions, or scaling up of (pilot) projects or activities in the next case, a larger one, or in other ways, a more encompassing case. For example, a local practitioner mentioned the strategy of replicating developed models of how to deal with threats from temporary glacial lakes in other places (GLA Prac-2 102, 503, 552).

Additionally, the interviewees often spoke about transferring knowledge from research to practice contexts. We found that this could only be partly understood as knowledge transfer between cases in TDR. A fine line exists between research and non-research activities, which has been drawn differently in the investigated projects: They span a spectrum ranging from practice projects with research components or advice from science to research projects with occasional participation from practice. Following our study’s goals, in this paper, we explicitly exclude knowledge that was stated to be used exclusively in practice and not in another TDR project (*Transfer to practice*). However, we include means of making knowledge available in practice or ways of organizing a transfer to practice if these means were declared to be in principle applicable in another TDR project.

### Transferable knowledge

The interviewees did state that in their view and with respect to their projects, there is knowledge that in principle is transferable. Knowledge generated in TDR that can be used in other cases accordingly includes the following: (1) Transdisciplinary principles, (2) transdisciplinary approaches, (3) systematic procedures, (4) product formats, (5) experiential know-how, (6) framings and (7) insights, data and information (Table 2). It encompasses both knowledge that interviewees reported to have transferred across projects, including all three pathways identified in the preceding section, and knowledge they considered transferable along these pathways. In the following, the different classes of transferable knowledge are described in more detail, and it is explained how they are distinguished from each other.

**Table 2:** Transferable knowledge identified

Transferable knowledge	Description	Examples
(1) TD principles	Nonspecific key overall fundamentals, learnings, concepts, rationales, values, or rules that can be followed; may be helpful when conducting TDR; may be thematically open or topic oriented	Take practitioners on board from the beginning of a project (NUC) to contribute their perspectives on what the key questions are; Need for researchers to really engage in the real-world context on a long-term basis (CON)
(2) TD approaches	Procedural in nature; give general guidance on how TDR processes can be shaped; represent particular schools of TDR; may be thematically open or topic oriented	Approach to setting up and organizing a TDR process in solution-oriented “learning watersheds” (WGOV); Approach to designing a locally relevant and well embedded early warning system (GLA)
(3) Systematic procedures	Adapted methodologies for specific analytical procedures or step-wise instructions providing information on how to collectively gain knowledge	Methodology for generating a hazard map based on the coupling of various models (GLA); Procedure for identifying barriers to technology adoption (TEC)
(4) Product formats	Product categories, in the sense of means and ways to communicate and use research results; intended for application in practice	Maps on land claims (LAND, GLA, ATL); Governance body for mitigating water conflicts (CON)
(5) Experiential know-how	Personal learnings, skills and experience gained; can only partly be passed on to other persons	Experiential insight for how to integrate knowledge in a diverse group (TEC); Confidence gained with respect to one’s ability to deal with a diverse stakeholder group (WGOV)
(6) Framings	Definitions and descriptions of issues, problems, or phenomena; reflecting particular perspectives; using various system boundaries; highlighting different aspects	Introduction of a concept for peace promotion in a business context (PEACE); Strength-based discourse in the field of public health as an alternative to problem-based framings (PREG)
(7) Insights, data and information	Concrete research results and outputs of various form and kind	Climate station observation data from Bolivia and south-eastern Peru (ATL); GIS data based information on transformations in land use (LAND)

#### (1) TD principles

*Transdisciplinary principles (TD principles)* are nonspecific but key overall fundamentals, learnings, concepts, rationales, values, or rules that can be followed and may be helpful when conducting TDR. Examples of TD principles include the importance of allowing practitioners to contribute their ideas and perspectives on what the key questions are to a project (Urban planning, URB), bringing them on board from the very beginning (NUC), designating local people as the project leaders (ATL), or involving them in the form of designated advisory or support groups (URB), as noted by a researcher:

“What I see as being very, very interesting and positive is the fact that we had this group of researchers and practitioners from the public and private sector that were able to say, ‘That is the relevant research question,’ or ‘That is something you’ve not talked about but I think you should consider it in your research agenda.’ So it’s framing the research question. We had to redraw the frame because of that contribution from this group. It was one point, and I think if I was doing it again I would definitely have a group like that again in any research project.” (URB Res 11)

Other TD principles refer to requirements, such as the recognition of the expertise and the values of the people involved in transdisciplinary processes (Low carbon technologies, TEC) or general procedural flexibility (Peace building, PEACE), as mentioned by a researcher:

“I think really it’s a little bit also learning by doing in the field, finding out what works and what doesn’t work. But then also writing it down, understanding the specific kind of changes that you need to make. And remember that sometimes in the field we were really having meetings and saying, ‘How do we go from here?’ We have this pre-given kind of methodology, but actually here now we can not apply it. ‘Hmm, how do we do this?’” (PEACE Res 83)

TD principles may be thematically open or topic oriented, for example, experience-based principles for natural resource management in conflict situations (Water conflicts, CON). The CON project leaders reported that they identified a set of success principles by reflecting on the TDR project experience. These principles include the need for researchers to really engage in the real-world context, as well as the requirement for long-term engagements.

#### (2) TD approaches

Like TD principles, *transdisciplinary approaches (TD approaches)* are unspecific, but procedural in nature, providing rather general guidance on how transdisciplinary processes can be shaped. Different approaches represent particular schools of transdisciplinarity. Examples found include approaches to joint problem identification with the affected community members or to setting up and organising transdisciplinary projects (e.g., Water governance, WGOV). TD approaches also include strategies for building alliances with regional partners, which may lead to partnerships that can be used for follow-up work. TD approaches may be thematically open or topic oriented. Examples for the latter include approaches to water management—setting up “learning watershed” schemes for planning and working on solutions (Water and land knowledge resources centers, WLRC)—or to dealing with glacier hazards through designing and implementing locally well-rooted early warning systems (GLA).

#### (3) Systematic procedures

*Systematic procedures* are specific analytical procedures or step-wise instructions providing information on how to collectively derive or gain knowledge. They include methodological developments, adaptations, or new contexts and ways in which such procedures are applied in TRD projects—as opposed to procedures and ways to communicate and use research in practice, which we call product formats. Examples are adapting a hydrological model to a karstic environment (WGOV) or scientific methodologies of generating hazard maps based on coupling various models (GLA), as noted by a researcher:

“the whole methodology [of] how to produce this (…) should be transferable, and then I would say [also] the way how it is handled then further on. But this, I think, is also context-specific to some degree. I mean, to some degree, you can transfer it, but it really depends on how the institutions work, how the whole disaster risk reduction or the emergency setup is taken. So this group, for instance, (…) [maybe] in Nepal, this could be quite different. But still, it might be an interesting event. In Peru, I think, it should be quite transferable, and maybe also in other Latin-American countries.” (GLA Res 49)

In NUC, the idea of conducting repeated monitoring surveys on people’s positions was developed (NUC Prac-1 76). Moreover, the basic democratic process of siting nuclear waste repositories, tailored to the way that a society works (NUC Prac-2 41-42), was stated to be transferable. Other project leaders listed systematic empirical procedures applied as part of theory-based analytical concepts. Working with the resilience framework was reported to have included an empirical step-wise procedure (PREG), for example. TEC developed a guideline describing a procedure for identifying, qualifying and quantifying barriers to technology adoption in collaboration with technology developers and users, using the example of the building sector:

“The guideline is a process for going with you in a three-step process. First, (…) you identify the most important (…) barriers, you identify the most important, or for you, most relevant aspects in these barriers. The second is you qualify these barriers so [that] you understand who creates the barrier, who is the one who owns the barrier, how big it goes within the system, which parts of the system are affected by these barriers, and what could be the effect on other technologies, and the third element is that you quantify [these barriers]. (…) What we also say is for the first two things you need to work with various groups of stakeholders, you cannot do it alone, sitting at your desk. You need to talk those who are involved in your system and this can be transferred to any other system. So if you want to understand what are the barriers for reducing deforestation (…), which is a completely different sector and it would be also geographically different, you could use the same approach: identify these barriers jointly with the different stakeholders and we explain in the guideline also which are the methods that you could use depending on the size of your system, and then you could qualify them with the stakeholders, you can find out which are the most relevant questions for this, and then you can go to quantification.” (TEC Res 73, 90)

Furthermore, two project leaders mentioned qualitative structured system analysis methods and scenario analysis, sensitivity assessment methodologies and stakeholder-related multicriteria assessment (PEACE, NUC). In NUC, the applied procedures served to identify stakeholders’ positions and preferred options, thus involving people in creating their future. Similarly, WGOV used a systematic procedure for participatory visioning, employing spatial thinking to create knowledge about desired futures by translating more or less abstract values into landscapes.

#### (4) Product formats

*Product formats* encompass different categories–or formats–of products, in the sense of means and ways to communicate and use research results, intended for use in practice. Transferability means that, in the next project, it may be worthwhile to develop the same or a similar kind of product to communicate or use TDR outputs in practice. Thus, this class of knowledge concerns the form of how a product for using research results is shaped, rather than the very product itself. Examples include various kinds of maps [Claims on land (LAND), GLA, ATL], policy briefs (PREG), organization structures (CON), or courses (LAND, GLA). Capacity-building courses in some cases built add-ons to the actual research while in other cases, they built an integral part of a transdisciplinary project. In the case of GLA, the researchers offered university courses because at local universities, the appropriate education was missing. Although capacity-building activities represent a transfer of scientific expertise into a practical learning context, the *idea* to incorporate it into TDR was considered transferable to the next project.

The above-mentioned product formats can be distinguished from those that provide instructions on knowledge transfer, such as manuals or guidelines that describe systematic processes and thus facilitate designing, shaping and applying the same or similar procedures in new contexts. Examples include recommendations for organizing climate station observations to support homogenizing data collection (ATL) or guidelines for designing participatory early warning system projects, including but not limited to developing or revising hazard maps, thus facilitating the application of a systematic procedure in a new case or context (GLA).

#### (5) Experiential know-how

*Experiential know-how* encompasses personal learning and experiences gained. Some interviewees reported that they transferred or could potentially transfer skills and experiences that they had acquired in one project to other projects. Such learning represents know-how about shaping TDR processes or experience-based insights with emotional components (e.g., how rewarding true collaboration with practitioners can be). In WGOV, a researcher reported that she gained confidence in her own ability to deal with a diverse stakeholder group, which she was able to transfer to other projects (WGOV Res). A practitioner experienced how important it was to act on equal footing with other stakeholders (NUC Prac-1 43, 44, 48). We found that experiential know-how as a subcategory is either embodied by a person such that it cannot easily be passed on or made accessible to and usable for others—i.e. implicit or tacit knowledge—or it consists of learning and personal insights that can be made explicit, shared and thus passed on to others to a large extent.

#### (6) Framings

Issues, problems, or phenomena occurring in a certain context can be defined and described in many different ways and from different perspectives, using various system boundaries and highlighting diverse aspects. Such descriptions are called *framings*. The framings identified in the 12 projects encompass specific understandings of and meanings ascribed to certain problematic phenomena, substantive concepts, theoretical assumptions and suggestions or ideas on how to conceptualize and structure an issue or phenomenon considered to be problematic. PEACE worked with the idea of relating the human rights discussions in businesses to peace promotion, which is not normally an issue in the private industry. An interesting detail is the fact that the project group introduced the corporate social responsibility concept in the context of low- and middle-income countries, which failed because their respective notions of responsibility were not shared.

“So I think it's important really to come away from the CSR [Corporate Social Responsibility] literature, as it is now, and really try to understand what peace-building elements are there before you apply these. And I think [since] we started to do more research on that, (…) we have a better understanding of the impacts that businesses can have in these kinds of environments. (…) So I think one thing is to really understand what does peace-building mean, more from a donor perspective, and then the second element is really to also be aware of the cultural differences in terms of what is actually the aim of business in society in general.” (PEACE Res 40, 42)

Furthermore, several project leaders listed theory-based and analytical alternative framings of problems among the knowledge that they considered transferable. Examples include strength-based discourse in the field of public health as an alternative to problem-based framings (PREG) or positive-impact approaches to conflict issues, replacing do-no-harm and risk mitigation rationales (PEACE). URB introduced a similar change in perspective by framing health in urban planning based on green space attractiveness instead of green space availability, as illustrated by a researcher:

“… just to give you an example, to come back to this sort of question, how much green space. I said, ‘Listen. That’s not the right question. The right question is, ‘What makes green spaces attractive for people to use them for health promoting behaviours?’” (URB Res 29)

#### (7) Insights, data and information

*Insights, data and information* represent concrete TDR project outputs and results which can be transmitted to some extent and under certain conditions. For instance, an ATL project leader listed climate station observation data from Bolivia and south-eastern Peru as potentially transferable. Through the research, higher quality data were provided.

#### Differentiation between the classes of transferable knowledge

Our results suggest that with classes (1)–(5) a major part of transferable knowledge in TDR consists of knowledge and learnings about TDR, about what is important in TDR, how it can be done, what is helpful to consider in TDR processes and how results and outcomes can be made usable for specific target audiences, i.e. knowledge on a meta level. They relate to conducting TDR, although they are not all completely procedural in nature but also include elements as regards content like, e.g. underlying values. Classes (1)–(3), transdisciplinary principles, transdisciplinary approaches and systematic procedures all relate to methodology but clearly differ with respect to their specificity and systematicity. One could argue that by definition, classes (1) and (2) encompass the other classes since they are of an overarching nature. However, the other classes, which are needed for the application of TD principles and approaches in doing research, do entail more specific information than the first two–which remain largely general. Therefore, it is useful to explicitly distinguish those from the TD principles and approaches as such.

Two out of the seven classes of transferable knowledge, namely (6) and (7) predominantly encompass case and context specific outcomes and learnings. We distinguish between framings that provide the conceptual basis, and insights, data and information as concrete research results.

### General characteristics of transferable knowledge

From the interviews, we conclude that while knowledge is in principle transferable, not every knowledge of a given class (e.g., not every specific framing or systematic procedure as such) is transferable to another case. According to our study, whether or not knowledge of a certain class is transferable is therefore not a “yes-or-no” question. The identified knowledge is stated to be transferable while being context sensitive to some degree (e.g., GLA). Transferability seems to be bound to certain circumstances or conditions. Even on the level of TD principles, transferability has its limits, as they relate to a certain school or understanding of TDR or co-production of knowledge. Furthermore, transfer was reported to sometimes involve adapting or further developing knowledge for use in a new context.

We found a set of characteristics of transferable knowledge that might be relevant for their actual transferability (see also Table 3):

- *Development level* (immature–mature). Depending on how strongly elaborated, tested and established the knowledge is, when transferred to a different case it either involves quite some experimentation or comes with a lot of clarity and explanatory power but may also entail more constraints with respect to transferability.
- *Specificity* (general–specific). Depending on how specific or general the knowledge is—and in which respect(s) —, it is primarily transferable to similar or completely different cases and contexts.
- *Adaptability* (rigid–flexible). Transferable knowledge can be flexible and can thus rather easily be adapted to a new case and context, whereas if rigid, knowledge must be transferred as it is.
- *Complexity* (simple–complex). Encompassing knowledge that conceptualizes many elements and aspects of a case requires all these elements to be taken into account in the new case.

**Table 3:** General characteristics of transferable knowledge: examples

Characteristic of transferable knowledge (Expression)	Examples	Class of transferable knowledge	Expression of characteristic
Development level (from immature to mature)	Using the concept of peace promotion in a business context as a new type of framing (PEACE)	Framing of issues	*Immature*: peace promotion turned out not to be an established concept in the studied business context and was thus immature when used in the TDR project PEACE
	Process of setting up an early warning system for risks related to glacial retreat, i.e. glacial lagoons involving stakeholders (GLA)	Systematic procedures	*Partly mature*: well-developed elements regarding technical infrastructure on the one hand and social involvement on the other hand were combined to something new
Specificity (from general to specific)	Way of setting up, discussing and organizing TDR as scientific work and interaction with stakeholders (WGOV)	TD approaches	*Rather general*: the approach can be used for regional development processes in general, but is restricted to basic democratic policy contexts
	Documenting land uses and land use rights in form of maps (LAND)	Product formats	*Quite specific*: specific with respect to the issue of differing perceptions on land use rights, e.g. land protection regulations conflicting with actual land use (e.g. through smallholders) and granted land use concessions
Adaptability (from rigid to flexible)	Involving stakeholders in form of an group accompanying the project (URB)	TD principles	*Very flexible*: the principle gives a general idea of what to do. Key elements can be determined flexibly, e.g. discussing or framing research questions; or project plans with respect to who to address with the knowledge
	Improved climate station observation data from Bolivia and south-eastern Peru (ATL)	Insights, data and information	*Rigid*: the data is static (rigid)
Complexity (from simple to complex)	Taking practitioners on board from the beginning of a project (NUC)	TD principles	*Simple*: the TD principle is simple and straight forward
	Model of water users associations (governance body), bringing together very different stakeholders (CON)	Product formats	*Complex*: such structures involve turning processes of resource degradation and social organization into well working participatory governance schemes

As our results suggest, whether or not knowledge is transferable requires a decision on a case-by-case basis since it seems to depend not only on the particularities of the case with its context but also on the knowledge’s specific characteristics. Several interviewees’ reasoning indicated that such characteristics play a role for considering knowledge transfers.

## Discussion

### Expanding the body of knowledge of TDR

An issue the interviewees stated was the question of how to make use of experience with knowledge co-production to improve and further develop the design of another or next TDR project. According to our rationale, such deliberation forms an essential part of and in turn enriches the body of knowledge of an academic field. The classes of transferable knowledge identified (see Table 2) seem to be (among) the basic elements of this body of knowledge. As elements of the body of knowledge, they can be collected, codified, stored, made accessible and imparted from experts to newcomers. These are key elements on which TDR scholars can capitalize. They need to be further developed in order to improve approaches to knowledge co-production in general and TDR in particular.

If the seven classes of transferable knowledge identified in this study belong to the body of knowledge of TDR, one might ask in how far TD scholars are more generally aware of them when discussing and further developing TDR. To approximate the answer, we discuss whether all seven classes are collected, characterized and made accessible, and if yes, how.

The discussion in the academic literature has been largely focused on *TD principles* and *TD approaches:* Attempts have been made to identify general TD principles or heuristics (Carew and Wickson 2010; Lang et al. 2012; Norstrom et al. 2020; Pohl and Hadorn 2007; Reed et al. 2014; Tapio and Huutoniemi 2014), to specify them for a particular context like the global south (van Breda and Swilling 2019) or for a specific stage of TDR processes (Gaziulusoy and Boyle 2013; Pearce and Ejderyan 2020). Different schools employ and defend their TD approaches, for instance, by presenting their specific take on the TDR process and its challenges (Carew and Wickson 2010; Defila and Di Giulio 2001; Gibbs and Beavis 2020; Jahn et al. 2012; Lang et al. 2012; Pohl and Hadorn 2007; Scholz and Steiner 2015). Recently, also the strengths and weaknesses of different approaches to co-production were compared and discussed (Bammer et al. 2020).

The other five classes of transferable knowledge have been less visible in academic discussions. *Systematic procedures* have increasingly been collected in scholarly literature, online toolboxes and grey literature, however, not yet systematically and comprehensively. Some systematic procedures are collected as ‘methods’ or ‘tools’ for TDR or for co-production of knowledge in general. In the scholarly literature there are publications on specific tools (Mitchell et al. 2015; O’Rourke and Crowley 2013), and contributions that analyse specific systematic procedures (Hoffmann et al. 2017; Woltersdorf et al. 2019) as well as publications that introduce collections of methods (Bergmann et al. 2012; McDonald et al. 2009; Pohl and Wuelser 2019; Vogel et al. 2013). Most knowledge on systematic procedures is, with the primary aim of being used, collected and made accessible in online toolboxes, such as the Tools for Integration and Implementation Sciences, the Team Science Toolkit, or the td-net toolbox.

Systematic procedures are key to make TDR processes accessible to and comprehensible for others. Descriptions of systematic procedures are helpful to gauge the quality of a TDR project proposal or to better understand the result of a TDR project. More encompassing descriptions and collections of systematic procedures are needed to allow for them to be debated, tested, further developed and referred to as academically legitimated resources.

*Product formats* are key in TDR because of their importance for problem solving. To our knowledge, product formats so far have been widely underreported as an element of the body of knowledge of TDR. They are not systematically collected in the scholarly literature. We assume, this is because they are perceived even more down-to-earth and practice-driven than systematic procedures for research purposes. Existing online resources and grey literature on product formats focus on single products, but not on purposes they can fulfil. For instance, there are numerous online-resources on “how to write a policy brief”. We even identified two academic papers on the issue, both from the field of health (DeMarco and Tufts 2014; Wong et al. 2016). However, these contributions do not discuss the significance or compare formats of products created in TDR processes.

*Experiential know-how* is not discussed as an element of the body of knowledge of TDR. As far as it concerns personal skills and experience, it is rather discussed as a quality or disposition of persons who engage in TDR (Guimarães et al. 2019; Stokols 1998, 2014). With respect to transferability, we are only aware of Nagy et al. (2020, 153), who find—like us—that some transferable knowledge stays “embodied in actors themselves”. The fact that there is also substantial experience-based know-how that can be passed on to others seems to have been widely ignored in academic literature on TDR.

In TDR literature, *framings* have not been appropriately discussed in the sense of forming a part of the body of knowledge. Most discussions centre on the question whether there is a set of standard framings for sustainability problems. Referring to medicine, they are called “syndromes of global change” (Lüdeke et al. 2004; Schellnhuber 1999) or, more recently, “Archetypes” (Oberlack et al. 2019), referring to philosophy and psychology. Such standard framings would help TD researchers to classify sustainability problems and to identify adequate ‘solutions’. However, this is not how framings were dealt with in the projects we studied. Here, framings of problems were used directly and as tools, for instance to understand different stakeholders’ viewpoints through a system perspective (NUC) or to look at an issue from a different perspective by introducing the concept of peace promotion in a business context (PEACE).

We consider framings of problems—and the theoretical ‘lenses’ they provide through which to look at and structure a phenomenon—to be an element of the body of knowledge of TDR that deserves more attention. Questions to be discussed are: Does TDR require framings to be grounded in a specific worldview, for instance, a general systems perspective (Bammer 2013) or a social-ecological systems perspective (Jahn et al. 2012; Ostrom 2009; Schuttenberg and Guth 2015; Stokols et al. 2013)? Could sustainability challenges be categorized and assigned to specific sorts of framings of problems? Or are framings mainly a tool to look at a problem in a fresh way? And if so, how can framings adequately account for the complexity of problems and the diversity of perspectives on those?

*Insights, data and information* are key elements of what TDR delivers for a specific case and context. They have not been discussed as an element of the body of knowledge of TDR, however. Since only very few examples were given in this study, it remains unclear at this stage whether and in what form this class of transferable knowledge forms a useful part of the body of knowledge. Currently, discussions rather focus on what to consider when concretely transferring knowledge of that class between specific cases: How comparable are the two cases and contexts (Adler et al. 2018)? And who from each case and context should be included in the process of transfer (Nagy et al. 2020)?

## Conclusions

We empirically analysed what knowledge researchers and practitioners consider to be transferable from one TDR project and case to another. Our qualitative, in-depth study of 12 projects indicates that researchers and practitioners regard a broad range of knowledge as transferable that we have analysed and structured in seven distinct classes. Our results furthermore show that this transferable knowledge holds certain general characteristics that are decisive for actual transferability in individual cases. We infer from our results that the identified transferable knowledge classes with their crosscutting characteristics belong to or maybe even largely constitute the body of knowledge of TDR. However, a closer look at the scholarly literature on TDR reveals that apart from TD principles and TD approaches, this body of knowledge lacks considerable substance. In order to take knowledge co-production in TDR a step further, efforts in developing and critically discussing transferable knowledge of the other classes, foremost systematic procedures, product formats and framings may be worthwhile.

In view of the first set of general characteristics of transferable knowledge identified (development level, specificity, adaptability, complexity) we furthermore see substantive need for more research on how specific characteristics hinder or help transferability of knowledge in TDR, and under which circumstances.

Last but not least, we would like to point out that our findings are also situated knowledge. That is, they come with open questions and possible limitations grounded in our study design. First, we analysed 12 Swiss TDR projects and our findings are limited to the investigated projects and possibly represent the thinking of a particular subcommunity of TDR. Second, the experiences of individuals are not the only source of knowledge when it comes to discover elements of the body of knowledge of TDR. The more established TDR becomes, relevant sources will also include publications on TDR theories and case studies or training courses introducing students to TDR, for example. Finally, we looked for knowledge TDR project leaders transfer and thus do not specify in how far such knowledge—and which of the identified classes—is exclusive for TDR. This is a question that could be interesting for further research, e.g. by comparing bodies of knowledge of TDR and other types of research.
